# Direct Delivery of High-Concentration Therapeutics for Refractory Chronic Rhinosinusitis

**DOI:** 10.7759/cureus.89251

**Published:** 2025-08-02

**Authors:** Svitlana Levytska, Donald F Schomer, Peter Baptista, Dawid Schellingerhout, Ali S Haider, Harry Ross

**Affiliations:** 1 Department of Otolaryngology, Chernivtsi Medical College, Chernivtsi, UKR; 2 Department of Neuroradiology, MD Anderson Cancer Center, Houston, USA; 3 Department of Otolaryngology, Al Zahra Hospital, Dubai, ARE; 4 Department of Radiology, MD Anderson Cancer Center, Houston, USA; 5 Department of Neurosurgery, Jones Graduate School of Business, Rice University, Houston, USA; 6 Department of Emergency Medicine, Chernivtsi Medical College, Chernivtsi, UKR

**Keywords:** allergy, chronic rhinosinusitis, refractory, sinus, therapeutic

## Abstract

Purpose: This study evaluates a novel route of administration for the treatment of refractory chronic rhinosinusitis (CRS).

Materials and methods: This prospective case series, conducted at a single institution, included 11 patients with refractory chronic rhinosinusitis (CRS) who had not responded to maximal medical therapy (MMT). Each patient received an antibiotic/steroid solution administered via sinus puncture and lavage (SP&L) into the anterior ethmoid air cells under topical anesthesia. SP&L procedures were performed on days one, three, and five of the study. Sino-Nasal Outcome Test-22 (SNOT-22) scores were recorded in the clinic before the first SP&L (day one), after the final lavage (day five), and at the final follow-up visit on day 30. Baseline SNOT-22 scores from day one were compared to those obtained on days five and 30 to assess symptom changes over time.

Results: Ten patients completed the study, with nine patients receiving durable relief. The median baseline SNOT-22 score was 31, the median day five SNOT-22 score was 6.5, and the median day 30 SNOT-22 score was 9. Mean±SD was 30.4±3.1, 8.9±5.9, and 11.1±9.5, respectively. Compared to baseline, the SNOT-22 scores at both day five and day 30 were highly significantly decreased (p < 0.0001).

Conclusion: Intra-ethmoid lavage of antibiotic/steroid solution appears to be an effective, easily performed, and safe procedure for CRS patients refractory to MMT.

## Introduction

Chronic rhinosinusitis (CRS) patients who fail maximal medical therapy (MMT) and are poor surgical candidates have limited treatment options available [1-3]. Topical nasal sprays may deliver insufficient concentrations, while oral formulations can result in undesirable systemic side effects [4-6]. A novel route of delivery is proposed that can offer greater local therapeutic concentrations than nasal sprays while reducing the unwanted systemic side effects of oral steroids and antibiotics.

Topical nasal therapies are both simple to administer and well tolerated. Unfortunately, the maximum deliverable dose is often measured in mcg/ml, which may be insufficient for refractory patients [4,7]. Systemic dosing of corticosteroids can deliver superior tissue concentrations but includes significant risks, including but not limited to sepsis, GI bleeds, cataracts, Cushing syndrome, and avascular bone necrosis. Systemic antibiotic overuse frequently results in GI dysfunction and vaginal candidiasis and has created an escalating drug resistance crisis [4,8]. For patient's refractory to MMT and not considered good candidates for surgery, alternative therapies are needed. Ideally, these would allow highly therapeutic localized sinus concentrations without systemic side effects while also enabling a variety of drugs by type and class to address the diversity of sinus disease.

For many diseases, direct injection at the site of pathology offers significant advantages over systemic or topical therapy. Practitioners in orthopedics, rheumatology, neurology, anesthesiology, and others regularly perform targeted injections with great success for a variety of maladies. A small volume of concentrated drug delivered locally can offer a greater therapeutic response while avoiding unwanted systemic side effects. This concept can readily be extrapolated to CRS by otolaryngologists. Historically, sinus needle puncture, both trans-oral and trans-nasal, for irrigation and aspiration, was easily performed, and it is postulated that this same route can be adapted for localized delivery of therapeutics, providing superior efficacy to pills or sprays in those patients' refractory to MMT [9,10].

Previous attempts at injection for CRS have targeted intra-tissue delivery to both polyps and turbinates, but neither addressed sinus disease directly. Reports of catastrophic ophthalmic issues thought to be secondary to inadvertent intravascular delivery of steroid particle emboli during turbinate injection limited further use and may have discouraged further evaluation of sinus cavity injection/lavage [1,4,5,11]. In contrast, topical lavage to a semi-enclosed space would not carry the same risk profile as intra-tissue/intravascular injection. 

Most existing injectables are optimized for either IV or intramuscular (IM) administration. These formulations have not been studied for use in the unique sinonasal environment [6,12,13]. Fortunately, a functional alternative with extensive relevant tissue exposure is readily available. A vast formulary of antibiotics and steroids exists as ophthalmic eye drops. Not only are ophthalmic formulations FDA/European Union (EU)/European Medicines Agency (EMA)-approved for the delicate corneal environment, but normal clearance of topical ocular delivery via the nasal cavity provides decades of sinonasal safety data. The use of ophthalmic formulations for sinus cavity lavage allows the evaluation of a novel route of administration utilizing existing formulations that benefit from extensive historical safety data [14,15]. The purpose of this study is to evaluate the feasibility and clinical outcomes for a novel route of administration for the treatment of refractory CRS. 

## Materials and methods

Patients

Patients with chronic rhinosinusitis (CRS) who presented to the academic clinical practices at Chernivtsi Medical College between November 1, 2019, and December 15, 2019, were eligible for inclusion in this case series. To qualify, patients were required to meet the diagnostic criteria for CRS as defined by the American Academy of Otolaryngology (AAO) and the 2012 European Position Paper on Rhinosinusitis and Nasal Polyps (EPOS). Additional inclusion criteria included a duration of CRS symptoms for at least six months, clinical and/or CT-confirmed pan-sinusitis with ethmoid sinus involvement, and failure of maximal medical therapy (MMT), meaning they remained significantly symptomatic despite receiving multiple courses of appropriate steroid, antibiotic, or allergy medications. Eligible patients also needed to have surgically intact sinuses, no gross anatomic deformities identified on physical exam or CT imaging, and a disease that was considered potentially responsive to either antibiotic or steroid therapy. Patients were excluded if they had Meltzer Grade III or IV nasal polyps (Grade I and II polyps were permitted) or were currently receiving oral antibiotic treatment at the time of enrollment.

Study design and intervention

A single-institution prospective case series was performed. MMT regimes at baseline were continued unchanged during screening, treatment, and follow-up. Screening CT scans were obtained and reviewed no more than 30 days before the first treatment. CT scanning assisted in the diagnosis, procedure planning, and exclusion of patients with gross anatomic abnormalities, tumors, and obvious non-inflammatory, noninfectious etiologies that would not be expected to be responsive to steroid or antibiotic therapy. Sinus puncture and lavage (SP&L) procedures were performed in an outpatient clinic setting under direct visualization using standard office supplies. All SP&L procedures were performed by a board-certified otolaryngologist (S.L.). With the patient seated in the exam chair, a topical decongestant was applied to the nasal cavity, and a local anesthetic was delivered trans-nasally to the anterior ethmoid bulla. Topical anesthesia consisted of 1% lidocaine soaked pledgets, applied to the anterior ethmoid mucosal surface. Patients one and two also received local injections of 1% lidocaine with epinephrine. Puncture of the anterior ethmoid cavity was performed with a 23-27G, five-inch spinal needle mounted on a 6cc syringe containing 3cc saline and 3cc air. Depth of penetration was limited to no greater than ½ the ethmoid diameter as measured on the screening CT scan. The syringe was aspirated with the needle in place, demonstrating an air flash and confirming placement in an air-filled space. The syringe was then gently flushed with observation of drainage at the osteomeatal complex (OMC), again indicating ethmoid cavity positioning of the needle. The combination of direct visualization, a tactile “pop” felt upon entry into the sinus, air flash on aspiration, and visualization of drainage with flush at the OMC provided strong confirmation of correct intra-ethmoid needle placement. Once needle placement was confirmed, the 6cc syringe was exchanged for a 1cc syringe preloaded with ophthalmic tobramycin/dexamethasone solution, of which 0.5cc was instilled intra-cavity, followed by needle removal. With tobramycin/dexamethasone lavage, fluid drainage at OMC was again observed in all patients, confirming intra-sinus cavity delivery. SP&L procedures were then repeated on the contralateral side. Bilateral SP&L procedures were performed on days one, three, and five for a total of three bilateral procedures. Sino-Nasal Outcome Test-22 (SNOT-22) scores were obtained in the clinic prior to day one and five lavages, and upon final follow-up on day 30 [16] (see Appendix, Figure 3).

Statistical analysis

Baseline SNOT scores at day one were compared to scores at five and 30 days, using Student's t-test with paired observations and significance at 0.05. SPSS version 25 (IBM Corp, Armonk, NY) was used for statistical analysis. 

## Results

Patients

Eleven patients were followed, with 10 completing the prescribed three SP&L's. Patient no. 4 had symptom recurrence at day 30, after which she underwent elective sinus surgery and was found to have fungal disease. However, no fungal disease was noted on pre-inclusion imaging. Patient no. 9 experienced a recurrence of migraine headache after the first lavage and elected to discontinue participation in the study. The headache was considered consistent with prior migraines, temporary and self-limiting. The median age of the 11 patients followed was 38 years. There were eight females and three males (Table 1).

**Table 1:** Patient demographics and clinical outcomes. SNOT-22: Sino-Nasal Outcome Test-22.

Patient	Age	Sex	SNOT-22 day one	SNOT-22 day five	SNOT-22 day 30
1	38	Female	28	7	5
2	29	Female	33	5	15
3	29	Female	32	4	7
4	38	Female	32	10	30
5	34	Male	30	4	2
6	62	Female	35	6	2
7	36	Female	28	18	22
8	22	Female	24	4	2
9	41	Female	43	-	-
10	66	Male	32	11	11
11	62	Male	30	20	15

Procedure results

**Figure 1:**
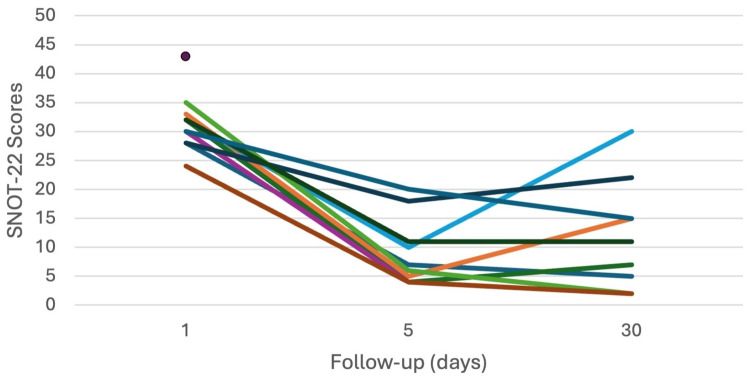
Spaghetti plot of individual SNOT-22 scores over time from baseline (day one) to last day of follow-up (day 30). SNOT-22: Sino-Nasal Outcome Test-22.

All patients tolerated SP&L well, with no discomfort reported from needle puncture after an adequate anesthetic block. Nine patients received durable relief. The median baseline SNOT-22 score was 31, the median day five SNOT-22 score was 6.5, and the median day 30 SNOT-22 score was 9. Mean±SD was 30.4±3.1, 8.9±5.9, and 11.1±9.5, respectively (Figure 1).

**Figure 2:**
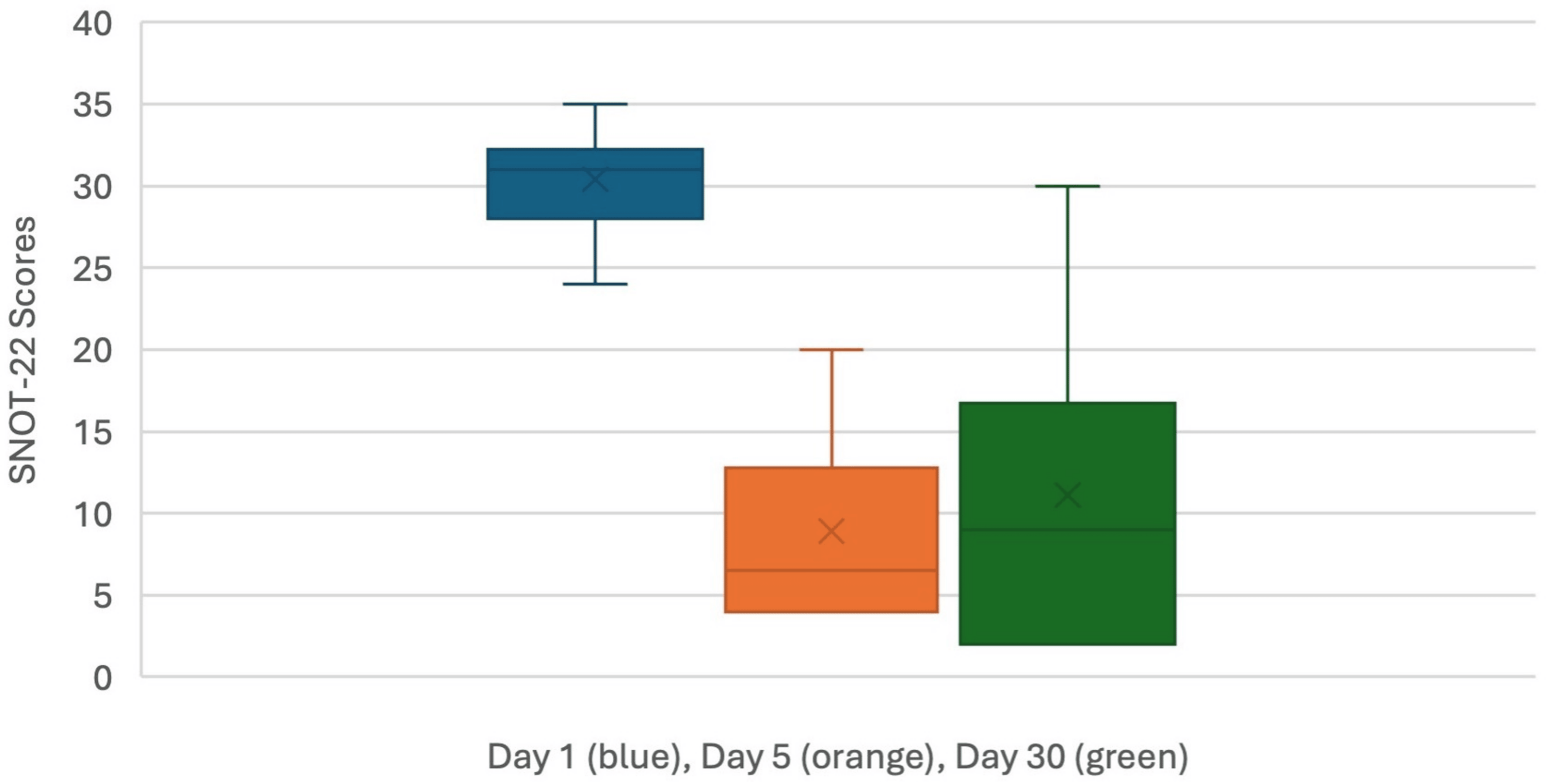
Boxplots comparing baseline SNOT-22 scores (blue) to scores at day five (orange) and 30 (green). There is a statistically significant decrease after treatment (p < 0.0001). X-axis scale is not intended to show progression over time. The caption at the bottom of the figure indicates days as identified by their corresponding boxplot color. SNOT-22: Sino-Nasal Outcome Test-22.

**Figure 3:**
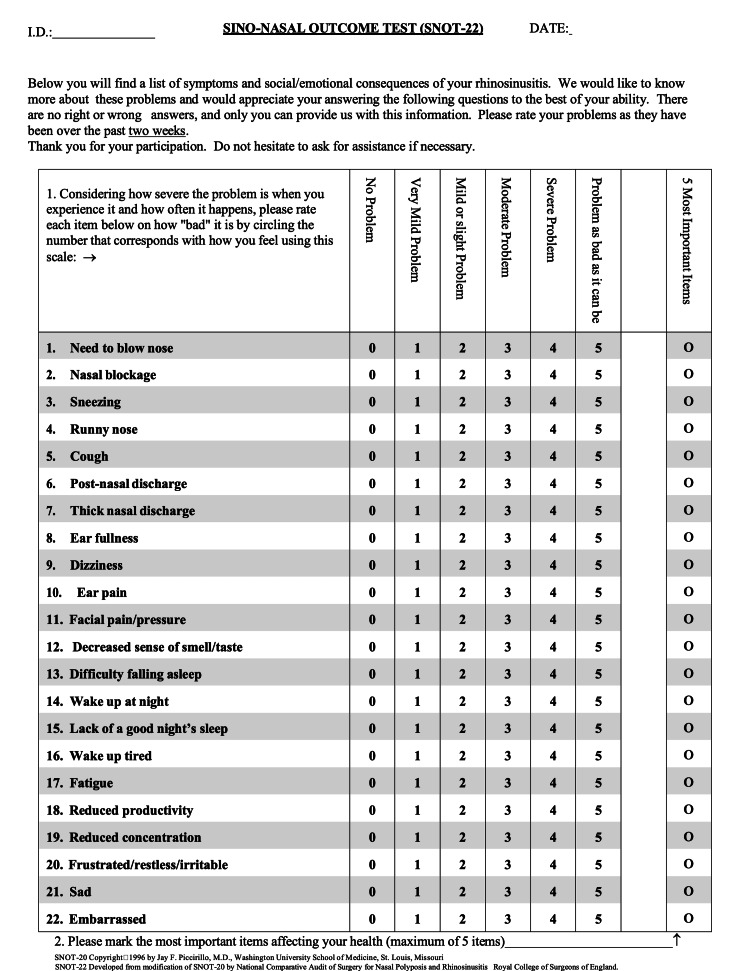
SNOT-22 questionnaire. SNOT-22: Sino-Nasal Outcome Test-22. SNOT-22 questionnaire obtained with permission from Washington University School of Medicine.

Compared to baseline, the SNOT-22 scores at both day five and day 30 were significantly decreased (p < 0.0001) (Figure 2).

Endoscopy on days one, three, five, and 30 revealed unchanged nasal tissue from baseline with no obvious trauma or change at the site of injection. 

## Discussion

Many CRS patients are well-managed with current treatment options. Alternatives would be useful for those who remain symptomatic despite nasal sprays and oral medications, or who are not good surgical candidates. In this study, anterior ethmoid puncture and lavage with FDA/EU EMA-approved ophthalmic antibiotic and steroid formulations provided significant relief and may offer an additional tool for otolaryngologists in the treatment of CRS. While a complete understanding of both the procedure and mechanism of action is not fully available at this early stage, theoretical observations and further questions can be considered.

Needle access for sinus aspiration or irrigation is a historical technique within the skill set of most otolaryngologists, and it is a small step from irrigation with saline to irrigation with active formulations [9,10]. As with any procedure, the anatomy must be understood and individual variations reviewed in assessing suitability for intervention. Most CRS patients will have had prior CT scans available, and if being considered for additional therapy, likely will have had updated recent imaging. With the increasing prevalence of in-office cone beam scanning, specific selected cuts can readily be obtained, offering immediate treatment assessment time [13,17,18]. 

Standard endoscopes, syringes, needles, pledgets, and lidocaine are commonly found in most exam rooms and are sufficient for this protocol. While it is often tempting to design or obtain newer, more exotic devices for each new procedure, as presented in this study, sinus puncture/lavage is straightforward and can be accomplished without the need for additional paraphernalia reducing cost, complexity, and crowding in exam rooms.

As presented, needle access of the anterior ethmoid bulla under direct visualization allows multiple levels of confirmation prior to medication delivery commitment. The ability to utilize fine-gauge needles for both placement confirmation and delivery is an advantage over large-bore needles or trocars. A clear tactile “pop” as one enters the bulla is easily appreciated within a few millimeters of tissue penetration. With correct placement and puncture of the sinus, aspiration should yield only air, and saline irrigation should demonstrate obvious drainage at the OMC/infundibulum. These three defined confirmatory steps provide comfort to both novice and experienced practitioners alike. At any stage, if placement is incorrect or unsure, the needle can be withdrawn and repositioned before drug delivery. For unusual anatomy or facilities with access, image guidance could offer a fourth confirmatory step. No limitations exist for patients who have either attempted or completed therapy on future traditional treatments or interventions. 

While topical nasal therapies are standard of care, the usual mcg/ml concentrations may not penetrate or deliver sufficient dosing within an intact sinus. Oral systemic dosing of antibiotics or steroids also yields only mcg/ml tissue concentration at the end-organ sinus tissue. Regardless of the nasal spray or oral route, mcg/ml may be insufficient for refractory patients. Direct delivery of mg/ml drug concentration within the sinus cavity has the ability to deliver log order higher concentration over existing spray or pill routes at the tissue level.

The semi-enclosed architecture of a surgically naïve sinus presents both challenges and opportunities for drug delivery. The purposeful choice of treating only surgically naïve patients enables prolonged drug retention compared to an open nasal cavity. In summary, ophthalmic formulations’ wide availability, extensive safety profile, and attractive concentration-to-volume ratio make them ideal candidates for sinus delivery.

As with any new therapeutic option, there is no one-size-fits-all. Understanding what differentiates good from bad patient candidates is critical to success and safety. Beyond initial anatomic considerations, different disease states require different considerations and would be expected to benefit from different treatment protocols. The availability of existing antibiotic steroid combinations greatly simplified early-stage work. Local topical delivery to the anterior ethmoid likely also provided expanded exposure to the OMC, infundibulum, and outflow tracts of both frontal and maxillary sinuses. Isolated sinus disease was not evaluated in this series, but lavage of other sinuses, particularly the maxillary's, could also be feasible. 

Rarely does a single dose of medication offer adequate response, and there was no reason to believe that patients in this series would respond to a single treatment either. In balancing the patient logistics of multiple treatments requiring multiple clinic visits against a not uncommon traditional seven to 10-day oral treatment regimen, a series of three QOD injections was chosen. The authors presumed that fewer injections might not have been sufficient to show a signal, while more would be too burdensome for compliance. Encouragingly, all patients showed a significant response by the third injection, lasting 30 days of follow-up. Further studies would be required to more accurately define the optimal number and spacing of treatments, as well as durability. If results are confirmed in larger studies, then the potential to modify formulations for even greater efficacy and reduced dosing would be desirable. 

CRS is often an inflammatory disease with intermittent infectious and allergic exacerbations. No single drug can treat all components or even subgroups within a single component. Existing ophthalmics provide a wide range of steroids, antibiotics, allergy, antivirals, anti-inflammatory, antifungal formulations, and newer biologics and leukotriene inhibitors available for use [11,12,19-21]. These cover, and in many cases exceed, the current formulary available to otolaryngologists, allowing appropriate and individualized patient treatment.

Patients with CRS are managed long-term and rarely cured. Symptoms may wax and wane, requiring repeated periods of aggressive treatment followed by intervals of mild or minimal intervention [7,22-24]. These severe exacerbations may well benefit from SP&L options. When inflammation is not covered by nasal spray or pills, local steroid delivery may provide rapid relief. Difficult infections or avoidance of systemic antibiotic side effects may be effectively addressed with antibiotic lavage. Uncontrolled allergy flares may be likewise addressed by delivering mast cell stabilizers. Targeted individualized SP&L could be the next step in treatment when traditional pills and sprays fail. 

As presented in this series, SP&L has low direct costs. Syringes and needles are inexpensive, and generic ophthalmics are often within the range of prescription pills and sprays. If SP&L provides greater relief and an acceptable duration, it is reasonable to expect total reduced prescription use, reduced office visits, and reduced missed work, which may additionally result in reduced patient and third-party expenses [2,25-27].

This study has several limitations that potentially bias the generalizability of these findings. Clinical findings may be due to the simple irrigation effect. This study had limited enrollment due to the few patients locally available who could be enrolled per the study inclusion/exclusion criteria. Representative CT scans were unable to be safely accessed at the time of manuscript preparation due to the escalation of military conflict in the region. Future studies would benefit from larger enrollment, longer follow-up, multi-center diversity, greater CT/endoscopy examinations, dosing/timing evaluation, formulation choices, as well as patient selection. Ophthalmic formulations are generally clear and watery, visually indistinguishable from saline. Ultimately, a randomized double-blind, saline/placebo-controlled study with crossover would offer significant statistical power. If efficacy can be shown, future studies should also evaluate optimal patient selection, dosage, timing of treatment(s), duration of response, and possible economic impact of this novel therapy.

## Conclusions

Patients with refractory CRS have limited treatment options. SP&L of the anterior ethmoid sinuses with ophthalmic tobramycin/dexamethasone formulation provided significant SNOT-22 improvement in refractory CRS patients. This procedure appears to be easily performed, well tolerated, and safe while providing a novel alternative for patients who have failed MMT and may not be surgical candidates. Further studies evaluating optimal patient selection, dosing, safety, and mechanism of action are warranted. Additional prospective data in larger populations can further support the adoption of this procedure.
